# AMPK exerts dual regulatory effects on the PI3K pathway

**DOI:** 10.1186/1750-2187-5-1

**Published:** 2010-02-18

**Authors:** Rong Tao, Jun Gong, Xixi Luo, Mengwei Zang, Wen Guo, Rong Wen, Zhijun Luo

**Affiliations:** 1 Department of Medicine, Molecular Medicine PhD Program, Boston University School of Medicine, 715 Albany Street, Evans 643, Boston, MA 02118, USA; 2 Department of Biochemistry, Boston University School of Medicine, 715 Albany Street, Evans 643, Boston, MA 02118, USA; 3 Department of Medicine, Section of Hematology and Oncology, University of Chicago, 5812 South Ellis Avenue, Chicago, IL 60637, USA; 4 Department of Medicine, Boston University School of Medicine, 610 Albany Street, Boston, MA 02118, USA; 5 Bascom Palmer Eye Institute, University of Miami, Miller School of Medicine, 1638 NW 10th Avenue, Miami, FL 33136, USA

## Abstract

**Background:**

AMP-activated protein kinase (AMPK) is a fuel-sensing enzyme that is activated when cells experience energy deficiency and conversely suppressed in surfeit of energy supply. AMPK activation improves insulin sensitivity via multiple mechanisms, among which AMPK suppresses mTOR/S6K-mediated negative feedback regulation of insulin signaling.

**Results:**

In the present study we further investigated the mechanism of AMPK-regulated insulin signaling. Our results showed that 5-aminoimidazole-4-carboxamide-1 ribonucleoside (AICAR) greatly enhanced the ability of insulin to stimulate the insulin receptor substrate-1 (IRS1)-associated PI3K activity in differentiated 3T3-F442a adipocytes, leading to increased Akt phosphorylation at S473, whereas insulin-stimulated activation of mTOR was diminished. In 3T3-F442a preadipocytes, these effects were attenuated by expression of a dominant negative mutant of AMPK α1 subunit. The enhancing effect of ACIAR on Akt phosphorylation was also observed when the cells were treated with EGF, suggesting that it is regulated at a step beyond IR/IRS1. Indeed, when the cells were chronically treated with AICAR in the absence of insulin, Akt phosphorylation was progressively increased. This event was associated with an increase in levels of phosphatidylinositol -3,4,5-trisphosphate (PIP3) and blocked by Wortmannin. We then expressed the dominant negative mutant of PTEN (C124S) and found that the inhibition of endogenous PTEN *per se *did not affect phosphorylation of Akt at basal levels or upon treatment with AICAR or insulin. Thus, this result suggests that AMPK activation of Akt is not mediated by regulating phosphatase and tensin homologue (PTEN).

**Conclusion:**

Our present study demonstrates that AMPK exerts dual effects on the PI3K pathway, stimulating PI3K/Akt and inhibiting mTOR/S6K.

## Background

AMP-activated protein kinase (AMPK) is a heterotrimeric enzyme consisting of an α catalytic subunit (α1, α2), and β (β1, β2) and γ (γ1, γ2, γ3) regulatory subunits [1]. The activation of AMPK occurs by binding of 5' AMP to the γ subunit and phosphorylation of T172 in the activation loop of the α catalytic subunit by upstream kinases such as LKB1 and CaMKK [1]. AMPK is activated in response to hypoxia, glucose deprivation, and muscle exercise, under which the AMP to ATP ratio is increased. In addition, AMPK activity is increased by certain hormones, such as leptin and adiponectin, and by pharmacological agents, including 5-aminoimidazole-4-carboxamide-1 ribonucleoside (AICAR), metformin, and thiazolidinediones. These agents are used in treating insulin resistance in animal models and/or in humans with type 2 diabetes and its complications [1].

AMPK exerts pleiotropic effects on cellular metabolism and has been emerged as a therapeutic target for the metabolic syndrome [2]. The activation of AMPK improves insulin resistance by stimulating glucose uptake and lowering blood glucose and lipid levels, whereas the activity of AMPK is suppressed in disorders associated with insulin resistance [2,3]. On the other hand, it increases fatty acid oxidation and inhibits fatty acid and protein synthesis, which is apparently opposite to the insulin action [3]. The latter often concurs with the scenarios when cells confront energy crisis. At molecular levels, complex relationship exists between the AMPK and insulin signaling pathways. For instances, AMPK has been reported to regulate IRS1 [4-6] and Akt/PKB [7-12], while insulin and Akt have negative impacts on AMPK activation [13-15].

The major effector of insulin is phosphoinositide 3-kinase (PI3K), which is activated by binding of the p85 regulatory subunit to specific sites on IRS1/IRS2 that are tyrosine-phosphorylated by the insulin receptor [16]. Activated PI3K phosphorylates phosphatidylinositol [4,5]-bisphosphate (PIP2) at 3' position, whereas phosphatase and tension homologue (PTEN) dephosphorylates this site and thus turns off the signal. Increased PIP3 recruits PDK1 and Akt to the plasma membrane whereby Akt is activated and becomes a major player of insulin action. An important modulator of inulin action is the mammalian target of rapamycin (mTOR), a member of the phosphoinositide kinase-related family that possesses exclusively protein kinase activity. mTOR functions in a mitogenic pathway downstream of PI3K and is activated by insulin and other mitogens in the presence of sufficient nutrients such as amino acids and glucose [17]. Activated mTOR regulates protein synthesis via phosphorylation of its targets, such as activation of S6 kinase 1 (S6K1) and inhibition of the initiation factor 4E binding protein (4E-BP1). In addition, mTOR and S6K1 have been shown to induce serine/threonine phosphorylation of IRS1 to attenuate signal flow to downstream effectors, and thus play a role in insulin resistance [18]. In contrast, when cells sense a shortage of nutrients, for instance, reduced cellular levels of glucose, or other stresses that deplete intracellular ATP, mTOR is inhibited and protein synthesis slows down, allowing ATP to be used for processes more critical to survival. This event is largely controlled by AMPK [3]. In fact, many studies have shown that the activation of AMPK leads to an inhibition of mTOR/S6K1 [19]. This occurs via phosphorylation of TSC2, an mTOR inhibitor [20,21], and Raptor, a scaffold protein of TORC1, essential for mTOR activity [22].

Despite the fact that AMPK activation enhances insulin sensitivity, the underlying mechanisms are not fully delineated. In the present study, we have investigated the interrelationship between AMPK and insulin signaling. Our results show that AMPK enhances activation of Akt by insulin, whereas it causes attenuation of mTOR/S6K1 signaling, both of which are beneficial to insulin action. Furthermore, our data indicate that AMPK activation also leads to increased phosphorylation of Akt through a novel mechanism dependent on PI3K but independent of PTEN.

## Results

### Effects of AICAR on insulin signaling

**Figure 1:**
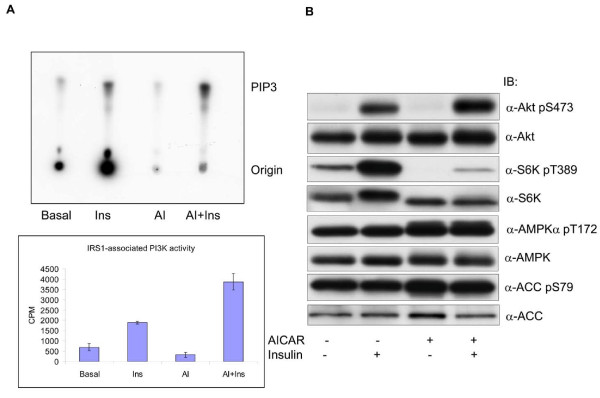
**Effect of AICAR on insulin-stimulated phosphorylation of the PI3K pathway**. 3T3-F442a preadipocytes were differentiated into adipocytes. A. The cells were treated with or without AICAR (AI) (1 mM, 2 h), followed by insulin (Ins) (100 nM, 15 min) and equal amounts of cell lysates were immunoprecipitated with antibodies against IRS1 and assayed on the associated PI3K activity. The radiolabeled PIP3 lipids were resolved on thin layer chromatography and exposed to X-ray film. The autoradiogram represents one of a triplicate experiment. The PIP3 spots were excised and counted in liquid scintillation. The graph denotes the average CPM of labeled PIP3 (n = 3, mean ± SD). B. In parallel to A, protein samples were run onto SDS-PAGE and blotted with antibodies, as indicated.

To evaluate the effect of AMPK activation on insulin signal transduction, 3T3-F442a adipocytes were treated with AICAR (1 mM, 2 h), followed by insulin (100 nM, 15 min), and IRS1-associated PI3K activity examined. As shown in Figure 1A, while AICAR treatment caused a slight inhibition of IRS1-associated PI3K activity at the basal level, it augmented the activity by almost 2-fold in the cells treated with insulin. Concurrently, pretreatment with AICAR enhanced insulin-stimulated phosphorylation of Akt at S473 by 90% (Figure 1B), which was accompanied by an increase in phosphorylation of GSK3 (data not shown). In contrast, insulin-stimulated phosphorylation of S6K1 was markedly suppressed by AICAR (Figure 1B). Similar results were obtained in differentiated 3T3-L1 adipocytes (data now shown)

### Dominant negative mutant of AMPK α1 subunit diminishes the effect of AICAR

**Figure 2:**
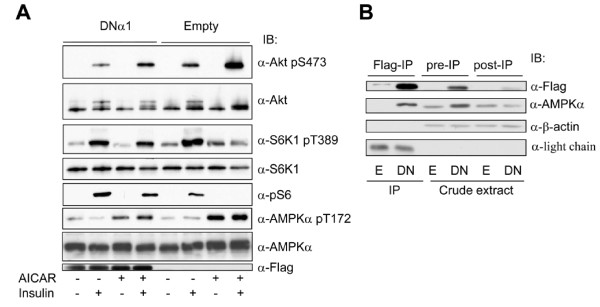
**Dominant negative mutant of AMPK α1 subunit suppresses the effect of AICAR**. 3T3-F44a preadipocytes were stably infected with lentivirus expressing a dominant negative mutant of AMPK α1 catalytic subunit (DNα1) tagged with Flag epitope or a control virus (Empty). A. The cells were treated with AICAR and insulin, as described for Figure 1, and assayed for immunoblot with antibodies, as indicated. B. Expression of DNα1 diminishes the endogenous counterpart. The cell extracts containing empty vector (E) or the AMPK mutant (DN) were immunoprecipitated (IP) with anti-flag antibody and equal amounts of pre-IP and post-IP extracts were blotted with anti-flag and anti-AMPK α1 antibodies, respectively. β-actin blot was used as an internal control.

To ascertain if the effects of AICAR are mediated by AMPK, we established stable cell lines in 3T3-F442a preadipocytes using a lentiviral system expressing a dominant negative AMPK α1 catalytic subunit (D157A mutation) [23]. We then assessed the effect of this mutant on AICAR-regulated phosphorylation of S473 on Akt in preadipocytes. One reason to use preadipocytes in this and the following experiments is that the ability of the preadipocytes expressing the mutant to differentiate into adipocytes is much greater than that in presence of the empty vector. As a result, it was difficult to compare the effects of insulin and AICAR in adipocytes differentiated from these two cell lines, owing to different expression levels of IRS1, insulin receptors, and AMPK (Data not shown). A second reason is that the expression of the dominant negative mutant somehow decreases the level of endogenous AMPKα subunits (Figure 2B), which seems to work as a true dominant negative interfering mutant. As shown in Figure 2A, the expression of the α1 mutant markedly inhibited insulin-stimulated Akt phosphorylation and also prevented the increase in the cells pretreated with AICAR. In contrast, mTOR/S6K1 signaling changed in an opposite direction, as manifested by the result that the α1 mutant prevented the reduction of S6K1 and S6 phosphorylation (Figure 2A). Thus, we conclude that the effects of AICAR on insulin signaling are mediated by AMPK.

### AMPK regulates Akt phosphorylation independent of IRS1 but dependent on PI3K

**Figure 3:**
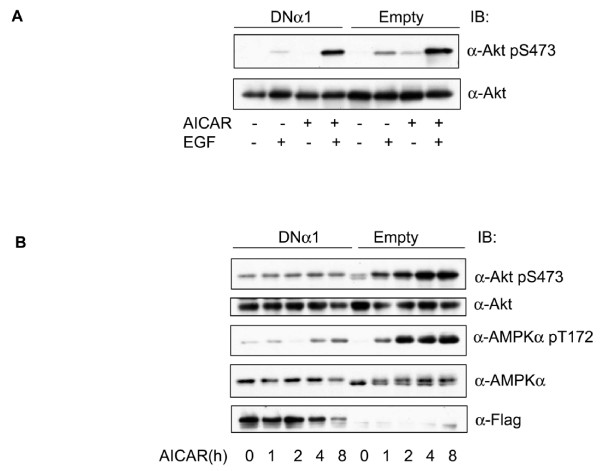
**AMPK promotes Akt phosphorylation independent of IRS1**. A. 3T3-F44a preadipocytes were treated with AICAR (1 mM, 2 h), followed by EGF (10 ng/ml, 15 min) and the cell extracts blotted with antibodies, as indicated. B. 3T3-F44a preadipocytes containing DNα1 or the empty viral vector were incubated with AICAR (1 mM) for different time, and the cell extracts blotted with antibodies, as indicated.

To seek after the factor that mediates AMPK-induced activation of Akt, we treated 3T3-F442a preadipocytes with EGF, which activates Akt independent of IRS1. As shown in Figure 3A, the dominant negative mutant of AMPK displayed a similar inhibitory effect on EGF-stimulated Akt phosphorylation. By comparing the phospho-signal intensity, we noticed that the Akt phosphorylation by EGF was much weaker than that by insulin (Data not shown). Longer exposure of Western blot allowed visualization of enhanced Akt phosphorylation when the cells were incubated with AICAR alone for 2 hours, which was blunted by the dominant negative AMPK mutant. This suggests that AMPK induces Akt activation independent of IRS1.

To confirm the direct effect of AMPK activation on Akt phosphorylation, we chronically treated the preadipocytes with AICAR and assessed the phosphorylation of Akt. As shown in Figure 3B, AICAR progressively increased phosphorylation of S473, reaching the maximum after 4 h. This was abrogated by expression of the dominant negative mutant of AMPK.

**Figure 4:**
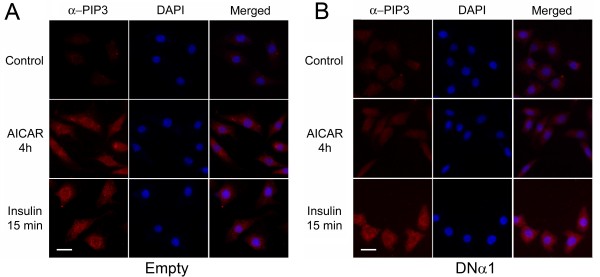
**AMPK activation stimulates production of PIP3**. 3T3-F44a preadipocytes bearing empty lentiviral vector (A) or the dominant negative mutant of AMPKα1 subunit (B) were treated with AICAR (1 mM, 4 h) or insulin (100 nM, 15 min). Cells were immunostained with anti-PIP3 monoclonal IgM antibody and counter-stained with DAPI for visualization of cell nuclei. Cells with empty viral vector accumulated PIP3 when treated with AICAR, as well as insulin (A). The AICAR induced PIP3 accumulation was not seen in the cells containing the AMPK mutant, whereas the effect of insulin was not affected (B). Scale bar: 63.4 μm.

Next, we examined if AMPK activation upregulates intracellular levels of PIP3. In doing so, the cells were treated with AICAR, as compared with insulin, and immunofluorescently stained with anti-PIP3 antibody. The data showed that the treatment of the cells with AICAR for 4 hours increased the abundance of PIP3 to a level similar to that induced by insulin (Figure 4A), whereas the effect of AICAR was almost completely suppressed by the expression of the dominant negative AMPK mutant (Figure 4B). Therefore, these results suggest that AMPK regulates Akt phosphorylation via regulating PI3K or PTEN, an event independent of IRS1.

**Figure 5:**
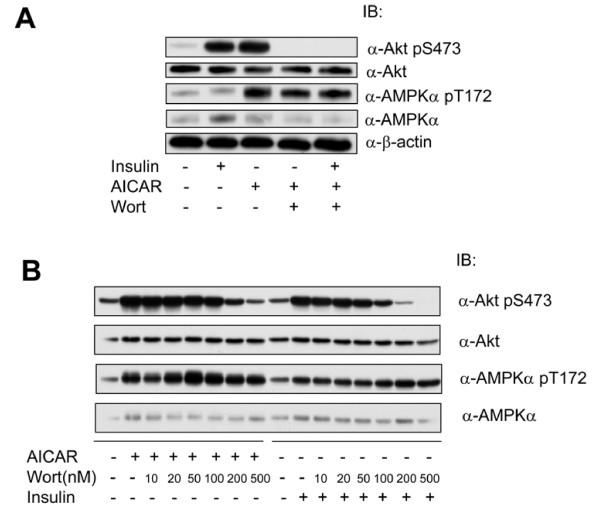
**Akt Activation by AICAR is suppressed by Wortmannin**. A. 3T3-F44a preadipocytes were incubated with Wortminnin (Wort, 0.5 μM, 30 min), followed by AICAR (1 mM, 4 h) or insulin (100 nM, 15 min). The cell extracts were blotted with antibodies, as indicated. B. 3T3-F44a preadipocytes were treated with Wortmannin at different doses, as indicated, for 30 min and followed by treatment with AICAR or insulin, as described for A. Cell extracts were blotted with antibodies, as indicated.

**Figure 6:**
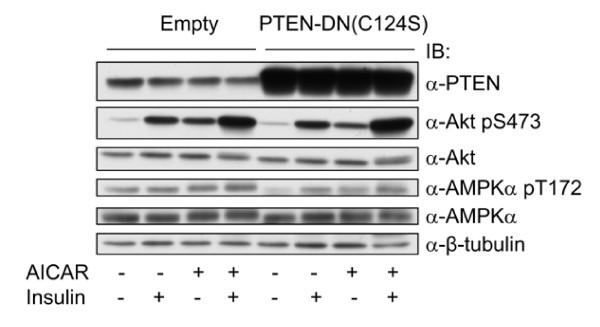
**Inhibition of PTEN does not affect AMPK-induced activation of Akt**. 3T3-F44a preadipocytes stably infected with the dominant negative PTEN (C124S) were treated with AICAR, insulin or together, as described for Figure 1, and the cell extracts were blotted with antibodies, as indicated.

We then asked if the effect of ACIAR could be blunted by Wortmannin, a specific inhibitor of PI3K. Thus, we treated 3T3-F442a preadipocytes with AICAR or insulin together with Wortaminnin. Figure 5 shows that Wortmainnin suppressed AICAR-induced Akt phoshorylation to an extent that is similar to its effect on insulin. To assess if PTEN is involved in AMPK regulation of Akt activation, we made stable cell lines expressing the dominant negative mutant PTEN C124S. As shown in Figure 6, the expression level of PTEN C124S reached approximately 10 times the endogenous protein; however, it did not display a significant effect on either insulin or AICAR-stimulated Akt activation. In a parallel experiment, the endogenous PTEN was knocked down with its shRNA by about 50%, which also did not yield an evident effect on Akt activation (data not shown). These results clearly exclude the possibility that AMPK regulates PTEN to impact on Akt activation. Therefore, they suggest that AMPK activates Akt via regulating PI3K.

## Discussion

In the present study we explored the mechanisms by which AMPK enhances insulin sensitivity. We observed that AICAR, a cell permeable metabolic precursor of the AMPK activator ZMP, enhanced insulin-stimulated Akt activation. In contrast, it impeded the ability of insulin to activate mTOR, a critical kinase for protein synthesis and cell growth. All these effects were suppressed by a dominant negative mutant of AMPK α1 catalytic subunit. Most intriguingly, our data provide new evidence that AMPK appears to activate Akt by regulating PI3K, instead of PTEN and IRS1.

It is well established that AMPK enhances insulin sensitivity and ameliorates insulin resistance. However, the underlying molecular mechanisms are not fully elucidated. The net effect of AMPK on insulin signaling is complex, with multiple targets involved depending on the cellular context. Although a few studies have shown that AMPK inhibits Akt [24], many found a positive effect [7,8,11,15,25]. Thus, AMPK activators such as AICAR, metformin or adiponectin enhance the effect of insulin on Akt activation, an event that is inhibited by overexpression of dominant negative mutant of AMPK [7,8,11,15,25]. A previous study has shown phosphorylation of S789 on IRS1 in vitro by AMPK and in vivo by AICAR in C2C12 myotubes [4], leading to an increase in IRS1-associated PI3K activity without changes in phosphorylation of tyrosine residues on IRS1 and its association with p85 regulatory subunit of PI3K. In contrast, Qiao and coworkers have reported that phosphorylation of S789 is associated with insulin resistance, which is not attributed to AMPK [5]. In addition, Tzatsos and Tsichlis have reported that AMPK activation induces phosphorylation of IRS1 at S794, leading to an inhibition of PI3K/Akt signaling [6]. The reason underlying this discrepancy is currently unclear. Although our study showed that AMPK activation enhanced insulin-stimulated IRS1-associated PI3k activity and subsequent activation of Akt, this probably occurs through regulation of PI3K. This is supported by three lines of evidence. First, Akt is directly activated by treatment of cells with AICAR in the absence of insulin and suppressed by Wortmannin. Second, AICAR increases the level of PIP3. Third, overexpression of the dominant negative mutant of PTEN does not seem to exert any effect on Akt activation, regardless that the finding is surprising to us, which is contrary to existing dogmas and probably reflects a cell-type difference. Our findings are in line with those of Ouchi et al, where it has been shown that adiponectin activates Akt in endothelial cells, which is dependent on AMPK and suppressed by PI3K inhibitor-LY294002 [7,8,11,15,25].

Hyperactivation of the mTOR-mediated pathway(s) has been observed in insulin desensitizing events and insulin resistant animal models [26-31]. This can be prevented or reversed by rapamycin [30,31]. Likewise, deletion of S6K1 alleles increases insulin sensitivity and protects mice against age- and diet-induced obesity [32]. The inhibitory effect of mTOR/S6K1 on insulin signaling correlates with increased S/T phosphorylation of IRS1 at three sites, S307, S636/S639. Recent studies have established that activated AMPK inhibits mTOR by phosphorylation of TSC2, a negative regulator, and Raptor, a positive regulator of mTOR, respectively [20-22].

## Conclusion

Our present study has demonstrated that AMPK enhances insulin sensitivity via at least two mechanisms; first, it is involved in direct regulation of PI3K and second, it inhibits mTOR/S6K to suppress negative feedback loop on the regulation of IRS1. While this latter mechanism has been recently defined, it is not clear how AMPK regulates PI3K. As there are several isoforms of PI3K, it will be interesting to determine which isoform of PI3K is the target of AMPK and how AMPK regulates its activity.

## Methods

### Materials

Antibodies against phospho- and nonphospho-proteins of Akt, S6K1, S6, AMPK, and ACC were purchased from Cell Signaling Technology (Danvers, MA). Antibodies for IRS1 were from Millipore (Billerica, MA). Mouse monoclonal IgM antibody against PIP3 was purchased from Echelon Bioscience (Salt Lake City, UT). Cyanine-3-conjugated goat anti-mouse antibody was purchased from Jackson Immunoresearch (West Grove, PA). Alexa Fluor 488 donkey anti-rabbit IgG was from Invitrogen (Carlsbad, CA). DAPI and trisacryl protein A beads were purchased from Pierce (Rockford, IL). PI(4,5)P_2 _was from Sigmaaldrich (St. Louis, MO). ^32^P-γ-ATP was from PerkinElmer (Fremont, CA).

### Cell culture

3T3-F442a and 3T3-L1 fibroblasts were grown and differentiated as described previously [33]. Cells were used 8 to 12 days after differentiation [34]. C4-2 prostate cancer cells were purchased from American Type Culture Collection and cultured in RPMI1640 containing 10% fetal bovine serum in 5% CO_2 _incubator.

### Plasmid DNA construction and transfection

The cDNA for dominant negative mutant of human AMPK α1 catalytic subunit was kindly provided by Dr. Carling [23] and cloned by PCR into a lentiviral vector where a flag epitope was added to the amino terminus of the α1 mutant. Lentivirus was prepared and the cells infected as described previously [35,36]. The cDNA for PTEN (C124S) in pSG5L (Addgene, Cambridge, MA) was digested with BamHI and EcoRI and subcloned into the same Lentiviral vector as used for AMPKα1 (TET-OFF) [35,36].

### Immunoblot

Cell extracts were separated by SDS-PAGE and electrophoretically transferred to immobilon, as described previously [34]. The membranes were blocked and sequentially incubated with specific primary antibodies and second antibodies conjugated with horseradish peroxidase. Immunoreactive bands were visualized by ECL.

### Immunofluoresent staining

3T3-F442a cells were cultured on coverslips pretreated with poly-L-lysine. Immunostaining was carried out, as previously described [34]. In brief, the cells were fixed in 4% paraformaldehyde in PBS and then washed with PBS. For PIP3 staining, after blocked with 10% normal goat serum (NGS) for 30 min, samples were incubated with mouse anti-PIP3 monoclonal antibody at 1:100 dilution for 2 h and non-immune mouse IgM was used as a negative control. After washing 3 times with PBS, the samples were incubated with Cyanine-3-conjugated goat anti-mouse antibody at 1:500 dilution for 1 h. All antibodies were diluted in PBS containing 2% NGS. After immunofluorescent staining, the cells were also counterstained with DAPI (0.5 μg/ml for 5 min) to detect the nuclei and the coverslips were mounted in spectrometric grade glycerol and sealed with nail polish. Fluorescent images were taken under Deltavision microscope (Applied Precision, Issaquah, Washington).

### Assay on PI3K activity

The assay was performed according to a protocol previously described [34]. Briefly, cell extracts were immunoprecipitated with antibodies against IRS1 preimmobilzed in trisacryl protein A beads. The immunocomplex was incubated with PI(4,5)P_2 _and ^32^P-γ-ATP. The labeled products were extracted, separated by thin-layer chromatography and exposed to X-Ray film. The PI(3,4,5)P_3 _spots were excised and measured by scintillation counting.

## Abbreviation list

ACC: acetyl coA carboxylase; AICAR: 5-aminoimidazole-4-carboxamide 1-D-ribonucleoside; AMPK: 5'AMP-activated protein kinase; Akt: protein kinase B; CaMKK: calmodulin-dependent protein kinase kinase; EGF: epidermal growth factor; IRS1: insulin receptor substrate-1; PAGE: polyacrylamide gel electrophoresis; PIP3: Phosphatidylinositol (3,4,5)-trisphosphate; PI3K: phosphoinositide 3-kinase; PDK1: Phosphoinositide-dependent kinase-1: PTEN: phosphatase and tension homologue; mTOR: mammalian target of rapamycin; TORC: mTOR complex; S6: ribosome S6; S6K1: ribosome S6 kinase 1.

## Competing interests

The authors declare that they have no competing interests.

## Authors' contributions

RT carried out major experiments including Western blots, establishing stable cell lines, and immunostaining. JG carried out Western blot analysis on differentiated 3T3-F442a adipocytes and PI3K assay. XL initiated this project and carried out initial experiments by establishing stable cell lines and Western blot analysis. MZ carried out initial Western blot. WG participated in designing and performing experiments. RW conceived of the study and formatted all figures. ZL is the corresponding author responsible for experimental design and coordination. All authors read and approved the final manuscript.
